# BV2 Microglial Cell Activation/Polarization Is Influenced by Extracellular Vesicles Released from Mutated SOD1 NSC-34 Motoneuron-like Cells

**DOI:** 10.3390/biomedicines12092069

**Published:** 2024-09-11

**Authors:** Elisabetta Carata, Marco Muci, Stefania Mariano, Elisa Panzarini

**Affiliations:** Department of Biological and Environmental Sciences and Technologies, University of Salento, 73100 Lecce, Italy; elisabetta.carata@unisalento.it (E.C.); marco.muci@unisalento.it (M.M.); stefania.mariano@unisalento.it (S.M.)

**Keywords:** amyotrophic lateral sclerosis, BV2 microglia cells, extracellular vesicles, neuroinflammation, mutated SOD1 NSC-34 cells

## Abstract

Microglia-mediated neuroinflammation is a key player in the pathogenesis of amyotrophic lateral sclerosis (ALS) as it can contribute to the progressive degeneration of motor neurons (MNs). Here, we investigated the role of mSOD1 NSC-34 MN-like cell-derived extracellular vesicles (EVs) in inducing the activation of BV2 microglial cells. NSC-34-released EVs were isolated by culture medium differential ultracentrifugation to obtain two fractions, one containing small EVs (diameter < 200 nm) and the other containing large EVs (diameter > 200 nm). BV2 cells were incubated with the two EV fractions for 12, 24, and 48 h to evaluate 1) the state of microglial inflammation through RT-PCR of IL-1β, IL-6, IL-4, and IL-10 and 2) the expression of proteins involved in inflammasome activation (IL-β and caspase 1), cell death (caspase 3), and glial cell recruitment (CXCR1), and presence of the TGFβ cytokine receptor (TGFβ-R2). The obtained results suggest a mSOD1 type-dependent polarization of BV2 cells towards an early neurotoxic phenotype and a late neuroprotective status, with an appearance of mixed M1 and M2 microglia subpopulations. A significant role in driving microglial cell activation is played by the TGFβ/CX3CR1 axis. Therefore, targeting the dysregulated microglial response and modulating neuroinflammation could hold promise as a therapeutic strategy for ALS.

## 1. Introduction

Microglial cells, known as the macrophages of the central nervous system (CNS), have long been suspected as key players in neurodegenerative diseases due to their capability to secrete either beneficial or harmful molecules. However, if their role in neuronal degeneration during development is well-known, the involvement in neurodegenerative diseases, including Amyotrophic Lateral Sclerosis (ALS), is not fully understood and still requires further investigation, in part because there is a shortage of experimental models that can accurately represent the complexity of ALS. A pathological microglial activation occurring before the clinical signs of the disease has been observed in the brain and spinal cord of both ALS patients and mouse models [1].

The ALS neurodegeneration is caused by an elective loss of upper and lower motor neurons (MNs) in the brain and spine leading to paralysis and eventually death from respiratory failure. The vast majority of ALS cases (90%) have an unknown aetiology and are considered sporadic (sALS), and only a small proportion (10–15%) of cases are familial (fALS) and follow an autosomal dominant pattern of inheritance [2,3,4]. Several mutations in more than 40 genes have been identified and associated with the onset of the disease [5], among them, mutations in the Cu/Zn Superoxide dismutase 1 (Cu/Zn SOD1) gene were the first to be identified and linked to the disease. At the cellular level, this mutant phenotype causes toxic protein aggregates, mitochondrial dysfunction, oxidative stress, and neuroinflammation, features observed in both ALS mouse models and ALS patients [6,7,8,9].

Over the years, evidence of the activity of non-neuronal cells in the development of the disease led to the focus of interest on the involvement of non-cell autonomous pathways [10,11,12]. Under normal physiological conditions, non-neuronal cells, such as microglia, astrocytes, and oligodendrocytes, play a crucial role in protecting and maintaining the CNS. In particular, the dynamic interactions between microglia and neurons are pivotal in maintaining homeostasis and ensuring the proper functioning of a healthy brain. Specifically, the communication between neurons and microglia is essential for neuronal development, synaptic plasticity, angiogenesis regulation, programmed cell death, and immune responses [13]. The activation of the microglia component in response to pathological stimuli, such as protein aggregates and oxidative stress, may lead to the expression of a potentially harmful inflammatory phenotype [14,15]. Although an acute inflammatory response is necessary to restore lost brain homeostasis and allow tissue repair, the persistence of significant inflammatory stress over time could promote the onset of neurodegenerative events that subsequently affect synaptic functions, plasticity, and cognitive abilities, contributing to various brain disorders, including ALS [16,17]. These observations have brought the possible relationship between neuroinflammation and MN loss to the forefront.

Microglia account for approximately 20% of glial cells in the CNS. Usually, microglia exist in a resting state and play a role in regulating synaptic communication and reshaping neuronal connections [18]. When the CNS is infected or injured, they are activated and migrate toward the infection site to remove infected cells and release cytotoxins to combat infections. If microglia are overly activated, the released substances can be harmful to nearby healthy tissues. Typically, microglia are categorized into M1 microglia, which are classically activated, and alternatively activated M2 microglia. The inflammatory M1 subtype is induced by Toll-Like Receptors (TLRs) and the gamma interferon (IFN-γ) signalling pathway, leading to the release of various pro-inflammatory cytokines like NF-kB, IL-1β IL-6, TNF-α, and chemokines. Conversely, the neuroprotective M2 subtype enhances the expression of Arg-1, the production of growth factors, and the secretion of anti-inflammatory cytokines such as TGF-β and IL-10 [19]. This differentiation between M1 and M2 microglia highlights the complex regulatory functions these cells serve in neuroinflammation and neuroprotection within the CNS. The shift of M1/M2 phenotypes is associated with neurodegenerative diseases, and the possibility of appropriately modulating microglial phenotypes, enhancing anti-inflammatory properties, and inhibiting or reducing M1 toxicity could represent a promising therapeutic strategy for ALS.

The relationship between the disease and factors involved in neuroinflammation has attracted increasing interest over the years, resulting in a growing number of publications reporting interesting results [20,21]. Yet studies have also highlighted the need to elucidate the mechanisms of communication between neuronal and non-neuronal cells.

Both physical contact and the release of soluble factors play key roles in neuron–microglia communication under both normal and abnormal conditions. As factors involved in the intercellular exchange of molecules, extracellular vesicles (EVs) are emerging as key players in cell-to-cell communication. EVs are nanosized vesicles enclosed by a lipid bilayer membrane and secreted by most eukaryotic cells, capable of carrying cargoes of heterogeneous nature such as proteins, cytokines, lipids, and nucleic acids, including DNA, mRNA, microRNA (miRNA), and long non-coding RNA [22]. EVs can be classified based on their cellular origin or their chemical/physical characteristics: the latest “Minimal information for studies of extracellular vesicles” (MISEV) guidelines by the International Society for Extracellular Vesicles (ISEV) identify two types of vesicles based on diameter and biogenesis, small EVs (30–200 nm), or exosomes, and large EVs (200–1000 nm), or microvesicles/ectosomes [23]. Small EVs originate as intraluminal vesicles (ILVs) following membrane invagination within multivesicular bodies (MVBs) which fuse with the plasma membrane allowing the release of vesicles into the extracellular milieu. Large EVs are produced as a result of the outward budding of the plasma membrane [24].

In the CNS, microglia, neurons, astrocytes, and oligodendrocytes release EVs not only to facilitate intercellular communication but also to eliminate toxic substances, such as IL-1β, interferons, tumour necrosis factor (TNF), and pro-inflammatory cytokines, from the cytoplasm [25]. Moreover, a plethora of studies reviewed in [26] highlight the crucial role of mesenchymal stem cell (MSC)-derived EVs, particularly in providing neuroprotection through the regulation of neuroglial cells. MSCs are pluripotent stem cells with significant therapeutic promise for neurological disorders because of their regenerative properties, immune modulation, and capacity to regulate inflammation. In 2016, the potential of MSC-EVs to have a neuroprotective effect in an in vitro model of ALS was demonstrated for the first time [27].

A growing body of research has described the involvement of EVs in neurodegenerative diseases, including ALS [22,28]; however, the impact of EVs released by damaged MNs on microglia activation is still little known.

The effects of EVs on recipient cells strictly depend on the state of neurons. Exosomes are specifically released by cultured neurons [29] and can be taken up by microglia. Neuronal exosomes inhibit the proinflammatory activation of microglia (M1) through their cargo, particularly the microRNAs found in exosomes originating from spinal cord neurons [30]. Upon adding neuron-derived EVs to primary cultured rat microglia, a significant enhancement in microglial viability through the inhibition of apoptosis and a decreased expression of activation surface markers have been observed. Moreover, neuron-derived EVs mitigated the LPS-induced pro-inflammatory response in microglia, as evidenced by a reduction in gene expression of pro-inflammatory cytokines (TNF-α, IL-6, MCP-1) and iNOS, while also promoting an increase in the expression of the IL-10 anti-inflammatory cytokine [31]. In ALS-damaged neurons, altered gene expression leads to the dysregulation of the EV-packaged cargo that, upon transfer to other cells, disrupts recipient cell function. Recently, we demonstrated that mSOD1 NSC-34 MNs-like cells release a high number of vesicles containing inflammation-modulating molecules, efficiently taken up by Raw 264.7 macrophages which, in turn, polarize towards a mixed pro-inflammatory and anti-inflammatory phenotype [32]. Also, Pinto demonstrated that NSC-34 MNs-like cells released exosomes containing mSOD1 are taken up by N9 microglial cells leading to an increase in the expression of the IL-1β, TNF-α, MHC-II, and iNOS genes in N9 cells, and to a decrease in the phagocytic capacity of these microglial cells [33]. Yin and colleagues observed that the expression of miRNA-21-5p significantly increases in both neuronal PC12 cells and released exosomes. The EVs containing miRNA-21-5p are taken up by microglia BV2 cells, which respond by changing their polarization status and contribute to chronic neuroinflammation via secretion of IL-1β, IL-6, and TNF-α pro-inflammatory factors [34].

The goal of this study is to investigate the role of mSOD1 NSC-34 motor neuron-like cell-derived EVs in inducing the activation of BV2 microglial cells. In this in vitro system, small and large EVs isolated by ultracentrifugation from NSC-34 murine motor neurons transfected with some of the most common SOD1 mutations (A4V, G93A, G85R, G37R) were incubated with BV2 microglial cells for 12, 24, and 48 h to evaluate the state of microglial inflammation and the expression of proteins involved in inflammasome activation, cell death, and glial cell recruitment.

## 2. Materials and Methods

### 2.1. Cell Cultures and Treatments

The mouse motor neuron cell line NSC-34 was kindly provided by Dr. Alessandro Romano (IRCSS-Istituto San Raffaele, Milan, Italy). The cells were grown in Dulbecco’s modified Eagle’s medium High-Glucose (DMEM-HG) (Biowest, Nuaille, France) supplemented with 10% heat-inactivated fetal bovine serum (FBS) (Biowest, Nuaille, France), 2 mM L-glutamine (Biowest, Nuaille, France), 100 U/mL penicillin and streptomycin (Sigma-Aldrich, St. Louis, MO, USA), in a 5% CO_2_ humidified atmosphere at 37 °C. Cells were passaged every 2 to 3 days before reaching confluence.

NSC-34 cells (35 × 10^3^/cm^2^ for each mutation considered) were transiently transfected into serum-free OPTI-MEM medium (Gibco, Whaltam, MA, USA) using Lipofectamine 2000 (Invitrogen, Whaltam, MA, USA) according to the manufacturer’s protocol, as reported in [30]. Briefly, a solution was prepared containing 500 μL of OPTI-MEM and 7 μL of Lipofectamine 2000, and a solution containing 500 μL of OPTI-MEM and 2.5 μg of plasmid DNA. After 20 min at room temperature, the diluted DNA was added to the diluted Lipofectamine 2000 and incubated for 30 min at room temperature. The cells were incubated with the freshly prepared solution for 24 h. Under our conditions, the transfection efficiency of NSC-34 cells was 80%. The transfection medium was then replaced with a growth medium consisting of DMEM-HG, 10% heat-inactivated FBS, and 2 mM L-glutamine for 48 h. Then, the selection of positive clones and induction of differentiation of NSC-34 cells into motor neurons were performed using a differentiation medium consisting of DMEM-HG/HAM’S F12 (Biowest, Nuaille, France) (1:1), 10% heat-inactivated fetal bovine serum (FBS) (Biowest, Nuaille, France), 2 mM L-glutamine (Biowest, Nuaille, France), 100 U/mL penicillin and streptomycin (Sigma-Aldrich, St. Louis, MO, USA), 1 × non-essential amino acids (Biowest, Nuaille, France), 1 × G418 (Biowest, Nuaille, France), and 1 μM retinoic acid (Sigma-Aldrich, St. Louis, MO, USA). Cells were grown in the differentiation medium for 4 days before extracellular vesicle (EV) isolation. Before use, all culture media were centrifuged at 100,000× *g* at 4 °C overnight to remove EVs from the FBS.

The BV2 murine microglia cell line was kindly provided by Dr. Alessandro Romano (IRCSS-Istituto San Raffaele, Milan, Italy). The cells were grown in a medium containing Roswell Park Memorial Institute (RPMI 1640) medium (Biowest, Nuaille, France) supplemented with 10% heat-inactivated fetal bovine serum (FBS) (Biowest, Nuaille, France), 2 mM L-glutamine (Biowest, Nuaille, France), and 100 U/mL penicillin and streptomycin solution. The cells were passaged every 2 to 3 days until confluence was achieved.

For each experimental condition here reported, 3 × 10^5^ BV-2 cells were seeded in RPMI 1640 medium. After 24 h, the growth medium was replaced with a medium containing 2 × 10^6^ isolated large EVs (lEVs) or 2 × 10^6^ small EVs (sEVs), or a vesicle-deprived conditioned medium (free EVs CM), and the BV2 cells were cultured for 12, 24, and 48 h in a 5% CO_2_ humidified atmosphere at 37 °C. The morphological modifications of the BV2 cells (as detailed in the Results paragraph) were evaluated using a light microscope, Nikon Eclipse80i (Nikon, Tokyo, Japan), and the images were taken using a DXM 1200F camera (Nikon Digital Camera, Tokyo, Japan).

### 2.2. Plasmids

Plasmids used for transfection were purchased from Addgene (Watertown, MA, USA): pF146 pSOD1WTAcGFP1 (Plasmid #26407); pF147 pSOD1A4VAcGFP1 (Plasmid #26408); pF148 pSOD1G37RAcGFP1 (Plasmid #26409); pF149 pSOD1G85RAcGFP1 (Plasmid #26410); pF150 pSOD1G93AAcGFP1 (Plasmid #26411). Plasmids containing the SOD1 mutations were amplified using competent Escherichia coli DH5α transformed with CaCl_2_. The plasmidic DNA was purified using the HiPure Plasmid Filter Maxiprep Kit (Invitrogen, Waltham, MA, USA). In these plasmids, the mSOD1 is in the same frame with Green Fluorescent Protein (GFP) to assess the transfection efficiency of NSC-34 cells.

### 2.3. EVs Isolation

At the end of 4 days of differentiation, EVs were isolated from the culture medium of NSC-34 cells by differential ultracentrifugation using the Beckman Coulter Optima XE centrifuge (Beckman Coulter, Brea, CA, USA), as reported in [35]. Briefly, the culture medium was three times centrifuged (500 g 5 min, RT; 800 g 10 min, RT; 2000 g (20 min, RT) to remove cells and cell debris. The obtained supernatant was centrifuged at 20,000 g (20 min, 4 °C). The pellet was collected and represents the large EV-enriched fraction; the supernatant was firstly filtered through 0.22 µm filters (polyethersulfone filter units, Thermo Fisher Scientific, MA, USA) and then centrifuged at 100,000 g (70 min, 4 °C). The pellet represents the small EV-enriched fraction. The obtained supernatant represents free EVs CM. The EV amount of each fraction and the characterization of EVs were measured before the start of the experiments, as reported in [32] and detailed in Figure S1.

### 2.4. Electrophoresis

A total of 12, 24, and 48 h after treatment, BV2 cells were lysed using a RIPA Lysis Buffer System (Santa Cruz Biotechnology, Dallas, TX, USA), prepared according to the manufacturer’s protocol. Briefly, the cells were washed three times with sterile PBS, lysed on ice by adding lysis buffer, detached with a cell scraper, and the collected lysate was placed on a rotator for 30 min. The lysate was then centrifuged for 10 min at 13,000 RPM at 4 °C. The supernatant was collected, and the protein concentration was determined using the Bradford assay. A total of 12 μg of protein was separated by SDS-PAGE electrophoresis by using a Mini Gel Tank (Thermo Fisher, Kiryat Shmona, Israel) on polyacrylamide gels, Novex Tris-Glycine Mini Protein Gels, 10%, 1.0 mm WedgeWell format (Life Technologies Corporation, Carlsbad, CA, USA).

### 2.5. Western Blot Analysis

After electrophoresis, proteins were transferred to nitrocellulose membrane Power Blotter Select Transfer Stacks (Invitrogen, Kiryat Shmona, Israel) by using a Power-blotter Semi-Dry Transfer System (ThermoFisher Scientific, Waltham, MA, USA). The membranes were blocked with sterile PBS containing 0.1% (*v*/*v*) Tween-20 and 5% (*w*/*v*) fat-free milk powder for 2 h. The membranes were incubated with the primary antibodies on a shaker overnight at 4 °C. The primary antibodies used were as follows: anti-β-actin (1:3000, Invitrogen, Waltham, MA, USA), anti-TGF-βR2 (1:2000, Proteintech, Manchester, UK), anti-Caspase 1 (1:2000, Proteintech, Manchester, UK), anti-Caspase 3 (1:1000, Proteintech, Manchester, UK), anti-CX3CR1 (1:1000, Proteintech, Manchester, UK), anti-MIF (1:500, Proteintech, Manchester, UK), and anti-Il-1β (1:1000, Proteintech, Manchester, UK). After washing with sterile PBS containing 0.1% (*v*/*v*) Tween-20, membranes were incubated with horseradish peroxidase-conjugated secondary antibodies (1:5000, Invitrogen, Waltham, MA, USA). Blots were developed using ECL reagent (Immobilon Crescendo Western HRP, Merck Millipore, Darmstadt, Germany). Images were taken with a ChemiDoc MP Imaging System (Bio-Rad, Hercules, CA, USA), and densitometric analysis of the bands was performed with ImageLab 6.1 software (Bio-Rad, Hercules, CA, USA). β-actin was used as a housekeeping protein for normalization of protein expression.

### 2.6. RT-qPCR

After 12, 24, and 48 h, the treated BV-2 cells were washed three times with sterile PBS. RNA extraction was performed using TRIzol reagent (Invitrogen, Waltham, MA, USA), according to the manufacturer’s protocol. A total of 2 ng of RNA was retrotranscribed using the SuperScript IV VILO Master Mix with ezDNAse enzyme kit (Invitrogen, Waltham, MA, USA). A CFX ConnectTM Real-Time PCR Detection System (Bio-Rad, Hercules, CA, USA) and SsoAdvanced Universal SYBR Green Supermix (Bio-Rad, Hercules, CA, USA) were used for RTq-PCR analysis. The primer sequences (Eurofins Genomics, Ebersberg, Germany) were as follows: β-actin (forward 5′-TGAGAGGGAAATCGTGCGTG-3′; reverse 5′-TGCTTGCTGATCCACATCTGC-3′); IL-1β (forward 5′-TCCATGAGCTTTGTACAAGG-3′; reverse 5′-GGTGCTGATGTACCAGTTGG-3′); IL-4 (forward 5′-GGCTAACAGACATCTTTGCTGCC-3′; reverse 5′-CAGTGTCCTTCTCATGGTGGCT-3′); IL-6 (forward 5′-CCATCTGGATTCAATGAGGAGAC-3′; reverse 5′-CTCTGGCTTGTTCCTCACTACCTC-3′); IL-10 (forward 5′-GATGCCTTCAGCAGAGTGAA-3′; reverse 5′-GCAACCCAGGTAACCCTTAAA-3′). Relative expression was calculated by 2−ΔΔCt, and the results obtained for β-actin were used for normalization.

### 2.7. Statistical Analysis

Data are expressed as Means ± SD. Multiple comparisons were performed by two-way ANOVA. Comparisons between the two groups were performed using a student’s *t*-test (GraphPad Prism 7 software, GraphPad Software, San Diego, CA, USA). *p* < 0.05 were considered significant.

## 3. Results

### 3.1. Exposure of BV-2 Cells to Extracellular Vesicles Derived from mSOD1-NSC-34 Motoneuron-like Cells Induces Morphological Activation and/or Cell Death

**Figure 1:**
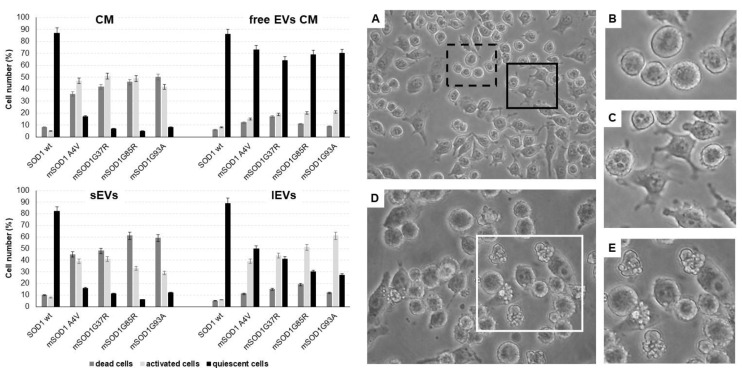
Morphological evaluation of BV2 cells cultured for 24 h in the conditioned medium (CM), vesicle-deprived conditioned medium (free EVs CM), small EVs (sEVs), and large EVs (lEVs) of mSOD1 NSC-34 MN-like cells. Left: cell number percentage of dead, activated, and quiescent BV2 cells measured by Nikon Eclipse phase contrast microscope. Right: phase contrast microscope images of BV2 cells. (**A**) BV2 cells challenged with lEVs from mSOD1G37R NSC-34 cells; (**B**) quiescent BV2 cells; (**C**) activated BV2 cells; (**D**) BV2 cells challenged with sEVs from mSOD1A4V NSC-34 cells; (**E**) apoptotic BV2 cells. Extracellular vesicles were isolated by differential ultracentrifugation allowing us to obtain two enriched fractions: small EVs (diameter ˂ 200 nm) and large EVs (diameter ˃ 200 nm). Bars: 10 μm. Plasmids used for transfection: pF146 pSOD1WTAcGFP1 (Plasmid #26407); pF147 pSOD1A4VAcGFP1 (Plasmid #26408); pF148 pSOD1G37RAcGFP1 (Plasmid #26409); pF149 pSOD1G85RAcGFP1 (Plasmid #26410); pF150 pSOD1G93AAcGFP1 (Plasmid #26411).

**Figure 2:**
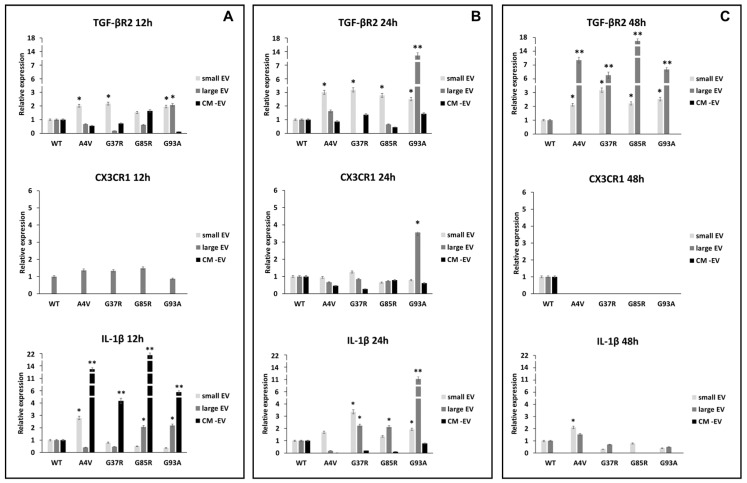
TGFβ/CX3CR1 axis regulation. BV2 cells were cultured for 12 (**A**), 24 (**B**), and 48 h (**C**) in the presence of vesicle-deprived conditioned medium (CM-EV), small EVs, and large EVs of mSOD1 NSC-34 MN-like cells. The expression of TGF-β type II receptor (TGFβ-R2), CX3C chemokine receptor 1 (CX3CR1), and interleukin 1β (IL1-β) is normalized to β actin and reported as a relative expression vs. SOD1WT NSC-34 MN-like cells considered as value 1. The values for all experimental groups reported in the histograms represent the means ± SD (n = 3) of three independent experiments; (*) *p* < 0.05 compared to the control SOD1WT; (**) *p* < 0.01 compared to the control (wt SOD1). Plasmids used for transfection: pF146 pSOD1WTAcGFP1 (Plasmid #26407); pF147 pSOD1A4VAcGFP1 (Plasmid #26408); pF148 pSOD1G37RAcGFP1 (Plasmid #26409); pF149 pSOD1G85RAcGFP1 (Plasmid #26410); pF150 pSOD1G93AAcGFP1 (Plasmid #26411). Images of representative blots are reported in Figure S2.

**Figure 3:**
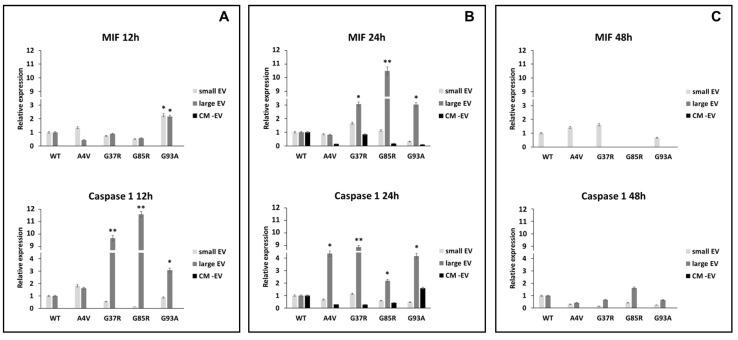
Expression of MIF and caspase 1. BV2 cells were cultured for 12 (**A**), 24 (**B**), and 48 h (**C**) in the presence of vesicle-deprived conditioned medium (CM -EV), small EVs, and large EVs of mSOD1 NSC-34 MN-like cells. The expression of MIF and caspase 1 is normalized to β actin and reported as a relative expression vs. SOD1WT NSC-34 MN-like cells considered as value 1. The values for all experimental groups reported in the histograms represent the means ± SD (n = 3) of three independent experiments; (*) *p* < 0.05 compared to control SOD1WT; (**) *p* < 0.01 compared to the control (wt SOD1). Plasmids used for transfection: pF146 pSOD1WTAcGFP1 (Plasmid #26407); pF147 pSOD1A4VAcGFP1 (Plasmid #26408); pF148 pSOD1G37RAcGFP1 (Plasmid #26409); pF149 pSOD1G85RAcGFP1 (Plasmid #26410); pF150 pSOD1G93AAcGFP1 (Plasmid #26411). Images of representative blots are reported in Figure S2.

As described in [32], mutated SOD1-NSC-34 motoneuron-like (mSOD1-NSC-34 MNs) cells release extracellular vesicles (EVs), both small (sEVs) and large vesicles (lEVs), in culture medium able to mediate the inflammatory process in Raw 264.7 macrophages. Here, the extracellular vesicles derived from mSOD1-NSC-34 MN-like cells are used to study the activation of BV2 microglial cells. Firstly, we evaluated the cell morphology of BV2 cells taking into account that, in physiological conditions, quiescent BV2 cells exhibit three distinct morphologies, i.e., rounded, bipolar/spindle, and multipolar shapes. Here we categorized BV2 cells based on the following three categories: (1) quiescent cells (rounded/fusiform/multipolar cells, with or without thin processes) (Figure 1A,B), (2) activated cells (amoeboid shape with flattened cell, swollen cell body, and/or thick retracted processes) (Figure 1A,C), and (3) apoptotic cells (shrunken and/or fragmented cells and blebs) (Figure 1D,E). Regardless of the type of mutation, BV2 cells cultured for 24 h with the conditioned medium (CM) of NSC-34 mSOD1 cells show an activated (about 50%) and apoptotic cell (about 40%) phenotype, unlike BV2 cells cultured with vesicle-deprived conditioned medium (free EVs CM) showing proliferative and/or quiescent morphology (about 70%). A high number of apoptotic figures are visible in the treatment with small EVs (sEVs) (about 50–60%), while the phenotype is a mix of quiescent (about 30–40%) and/or activated (40–60%) cells in the treatment with large VEs (lEVs) (Figure 1).

### 3.2. mSOD1-NSC-34 Motoneuron-like Cell-Derived EVs Regulate the TGFβ/CX3CR1 Axis in BV2 Cells

The maintenance of innate immunity in the brain is regulated by transforming growth factor-β2 (TGF-β2)-TGF-β type II receptor (TGFβ-R2)-CX3C chemokine receptor 1 (CX3CR1) signalling, which drives the activation of microglia [36]. The high expression of TGFβ-R2 downregulates the activation of CX3CR1 expression resulting in increased interleukin 1β (IL-1β) expression levels. Our results suggest that the TGFβ/CX3CR1 axis is regulated in a mutation superoxide dismutase 1 (mSOD1) type-dependent manner. As reported in Figure 2A and in Table 1, the BV2 cells exposed to small extracellular vesicles (sEVs) derived from mSOD1A4V-NSC-34 MNs-like cells rapidly after 12 h of exposure respond with expression levels of TGF-βRII equal to 2.026 ± 0.12 fold change vs. WT, with consequent IL-1β increase of 2.79 ± 0.14 fold change. The BV2 cells exposed to mSOD1G37R-NSC-34 MN-like-derived sEVs responded after 24 h of exposure with an expression of CX3CR1 equal to 1.26 ± 0.6 and TGFβ-R2 equal to 3.18 ± 0.12 fold change, with consequent IL1-β increasing of 3.36 ± 0.16 fold. Regarding the BV2 cells exposed to sEVs derived from mSOD1G85R-NSC-34 MN-like and mSOD1G93A-NSC-34 MN-like cells, we observed after 12 h of exposure an increase of TGFβ-R2, 1.529 ± 0.076, and 1.956 ± 0.99 fold change, respectively, but the consequent increase in fold change of IL-1β, 1.35 ± 0.068 and 1.928 ± 0.1, respectively, have been observed only after 24 h of culture (Figure 2B; Table 1). The significant increase in TGFβ-R2 expression starts after 24 h of exposure to sEVs for all mutation types (3 ± 0.1 for mSOD1A4V, 3.18 ± 0.16 mSOD1G37R, 2.79 ± 0.12 mSOD1G85R, 2.51 ± 0.15 mSOD1G93A) (Figure 2B; Table 1) and remains very high also after 48 h of culture in the presence of sEVs (2.11 ± 0.1 for mSOD1A4V, 3.18 ± 0.16 mSOD1G37R, 2.22 ± 0.13 mSOD1G85R, 2.53 ± 0.16 mSOD1G93A) (Figure 2C; Table 1). The exposure of BV2 cells for 12 h to large extracellular vesicles (lEVs) derived from mSOD1-NSC-34 MN-like cells does not show any type of regulation for all mutations considered (Figure 2A; Table 1). For the mSOD1A4V, mSOD1G37R, and mSOD1G85A mutations, an absence of regulation is confirmed also after 24 h of exposure, on the contrary, it is possible to observe an over-expression only for TGFβ-R2 after 48 h, 7.60 ± 0.4, 6.2 ± 0.29, 17.57 ± 0.9, and 6.85 ± 0.3 fold change vs. SOD1 WT for mSOD1A4V, mSOD1G37R, mSOD1G85R, and mSOD1G93A, respectively (Figure 2C; Table 1). BV2 cells exposed to lEVs derived from mSOD1G93A-NSC-34 MN-like cells after 24 h of exposure show significant increases in expression for CX3CR1, TGFβ-R2 and IL-1β (3.55 ± 0.16, 13.71 ± 0.66, and 11.09 ± 0.65 fold change vs. SOD1WT, respectively) (Figure 2B; Table 1). Finally, the exposure of BV2 cells to the vesicle-deprived conditioned medium (free EVs CM) shows a significant increase in IL-1β (13.61 ± 0.71, 4.17 ± 0.23, 21.95 ± 0.99, and 5.93 ± 0.3 fold change vs. SOD1WT for mSOD1A4V, mSOD1G37R, mSOD1G85R, and mSOD1G93A, respectively) only after 12 h (Figure 2A; Table 1). No type of regulation is observed for TGFβ-R2 and CX3CR1 in all mutations at any time considered (Figure 2A–C; Table 1).

### 3.3. mSOD1-NSC-34 Motoneuron-like Cell-Derived EVs Regulate the Expression of MIF and Caspase 1 in BV2 Cells

Lang et al. have described an interesting role for MIF (macrophage inhibitory factor) in the activation of the nucleotide-binding domain, leucine-rich–containing family, pyrin domain–containing-3 (NLRP3) inflammasome, which is involved in the activation of the IL-1α, IL-1β, and IL-18 cytokines; in particular, the activation of pro-IL-1β in IL-1β is mediated by caspase 1 [37]. In our results, we show that, after 24 h, the MIF protein is overexpressed in BV2 cells exposed to large extracellular vesicles (lEVs) derived from mSOD1-NSC-34 MN-like cells (3.07 ± 0.14, 10.78 ± 0.48, and 3.02 ± 0.1 fold change vs. mSOD1WT for mSOD1G37R, NSC-34mSOD1G85R, NSC-34, and mSOD1G93A, respectively) (Figure 3B; Table 1). The BV2 cells exposed to lEVs derived from mSOD1G37R, mSOD1G85R, and mSOD1G93A-NSC-34 motoneuron-like cells show high expression levels of caspase 1 protein of about 9.77 ± 0.51, 11.64 ± 0.53, and 3.08 ± 0.1 fold change vs. mSOD1WT, respectively, after 12 h of exposition (Figure 3A; Table 1). The same trend is confirmed after 24 h of culture (Figure 3B; Table 1); conversely after 48 h, in all conditions, we do not observe any modification in the expression of MIF and the caspase 1 (Figure 3C; Table 1). BV2 cells exposure to small extracellular vesicles (sEVs) and conditioned medium without EVs does not affect the regulation of MIF and caspase 1 at any time point (Figure 3; Table 1).

### 3.4. mSOD1-NSC-34 Motoneuron-like Cell-Derived EVs Regulate the Apoptosis via Caspase 3 in BV2 Cells

**Figure 4:**
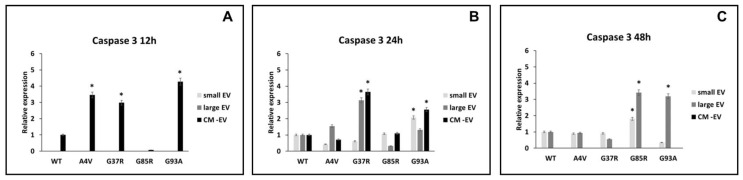
Expression of caspase 3. BV2 cells were cultured for 12 (**A**), 24 (**B**), and 48 h (**C**) in the presence of vesicle-deprived conditioned medium (CM-EV), small EVs, and large EVs of mSOD1 NSC-34 MN-like cells. The expression of caspase 3 is normalized to β actin and reported as a relative expression vs. SOD1WT NSC-34 MN-like cells considered as value 1. The values for all experimental groups reported in the histograms represent the means ± SD (n = 3) of three independent experiments; (*) *p* < 0.05 compared to control SOD1WT. Plasmids used for transfection: pF146 pSOD1WTAcGFP1 (Plasmid #26407); pF147 pSOD1A4VAcGFP1 (Plasmid #26408); pF148 pSOD1G37RAcGFP1 (Plasmid #26409); pF149 pSOD1G85RAcGFP1 (Plasmid #26410); pF150 pSOD1G93AAcGFP1 (Plasmid #26411). Images of representative blots are reported in Figure S2.

Based on the results achieved about caspase 1 and IL-1β and the morphological evaluation, we investigated the activation of apoptosis effector caspase 3 upon culture for 12, 24, and 12 h of BV2 with small extracellular vesicles (sEVs), large extracellular vesicles (lEVs), and vesicle-deprived conditioned medium (free EVs CM) derived from mutant superoxide dismutase 1 (mSOD1)-NSC-34 motoneuron-like cells. As shown in Figure 4A and Table 1, the level of caspase 3 is increased in microglial cells exposed for 12 h to free EVs CM derived from mSOD1A4V-NSC-34 MN-like cells (3.45 ± 0.16 fold change vs. SOD1WT), mSOD1G37R-NSC-34 MN-like cells (2.98 ± 0.13 fold change vs. SOD1WT), and mSOD1G93A-NSC-34 MN-like cells (4.27 ± 0.18 fold change vs. SOD1WT). After 24 h of exposure, the caspase 3 expression levels are increased in the BV2 cells exposed to lEVs and free EVs CM derived from mSOD1G37R-NSC-34 motoneuron-like cells, 3.13 ± 0.16 and 3.64 ± 0.16 fold changes, respectively, as well as in in the BV2 cells exposed to sEVs (2.07 ± 0.11 fold change vs. SOD1WT) and free EVs CM (3.56 ± 0.16 fold change vs. SOD1WT) derived from mSOD1G93A-NSC-34 MN-like cells (Figure 4B; Table 1). After 48 h, we observed high expression levels of caspase 3 in BV2 cells cultured with lEVs and free EVs CM derived from mSOD1G85R-NSC-34 MN-like cells, 1.80 ± 0.16 and 3.41 ± 0.16 fold change vs. SOD1WT, respectively, as well as in BV2 cells exposed to lEVs derived from mSOD1G93A-NSC-34 MN-like cells (3.19 ± 0.16 fold change vs. SOD1WT) (Figure 4C; Table 1). These results suggested that lEVs derived from mSOD1-NSC-34 motoneuron-like cells affect microglial cell apoptosis. After 12 h of exposure, no effects on caspase 3 are observed in BV2 cells with sEVs and lEVs. Moreover, no effects on the expression of caspase 3 are reported after 48 h of exposure in BV2 cells treated with free the EVs CM and sEVs, except for the BV2 cells exposed to sEVs derived from mSOD1G85R (1.80 ± 0.09 vs. SOD1WT) (Figure 4C; Table 1).

### 3.5. mSOD1-NSC-34 Motoneuron-like Cell-Derived EVs Modulate Polarization Status of BV2 Cells

**Figure 5:**
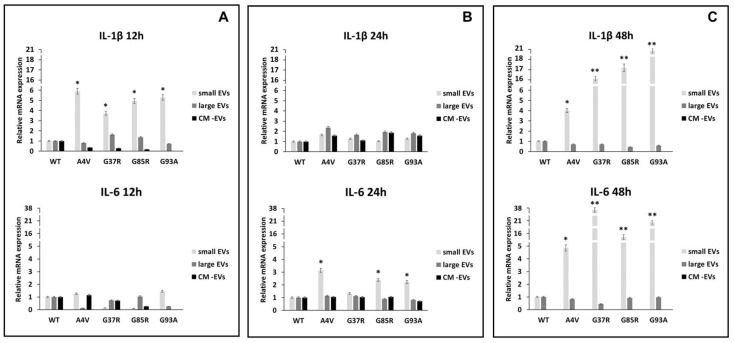
Polarization of BV2 microglial cells. mRNA levels of pro-inflammatory interleukin 1β (IL-1β) and interleukin 6 (IL-6) in BV2 microglial cells challenged for 12 (**A**), 24 (**B**), and 48 h (**C**) with vesicle-deprived conditioned medium (CM-EV), small EVs, and large EVs produced by mSOD1 NSC-34 MN-like cells. Data are normalized to β actin and are expressed as fold levels of mRNA of BV2 cultured with sEVs, lEVs, and free EVs CM vs. mRNA levels of SOD1WTNSC-34 MN-like cells considered as value 1. Values are means ± SD (n = 3); (*) *p* < 0.05; (**) *p* < 0.01. Plasmids used for transfection: pF146 pSOD1WTAcGFP1 (Plasmid #26407); pF147 pSOD1A4VAcGFP1 (Plasmid #26408); pF148 pSOD1G37RAcGFP1 (Plasmid #26409); pF149 pSOD1G85RAcGFP1 (Plasmid #26410); pF150 pSOD1G93AAcGFP1 (Plasmid #26411).

**Figure 6:**
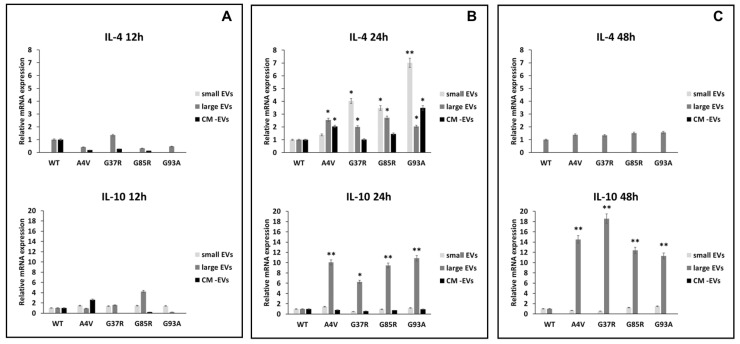
Polarization of BV2 microglial cells. mRNA levels of pro-inflammatory interleukin 1β (IL-1β) and interleukin 6 (IL-6) in BV2 microglial cells challenged for 12 (**A**), 24 (**B**), and 48 h (**C**) with vesicle-deprived conditioned medium (CM -EV), small EVs, and large EVs produced by mSOD1 NSC-34 MN-like cells. Data are normalized to β actin and are expressed as fold levels of mRNA of BV2 cultured with sEVs, lEVs, and free EVs CM vs. mRNA levels of SOD1WTNSC-34 MN-like cells considered as value 1. Values are means ± SD (n = 3); (*) *p* < 0.05; (**) *p* < 0.01. Plasmids used for transfection: pF146 pSOD1WTAcGFP1 (Plasmid #26407); pF147 pSOD1A4VAcGFP1 (Plasmid #26408); pF148 pSOD1G37RAcGFP1 (Plasmid #26409); pF149 pSOD1G85RAcGFP1 (Plasmid #26410); pF150 pSOD1G93AAcGFP1 (Plasmid #26411).

The polarization status of BV2 microglial cells challenged with mSOD1-NSC-34 motoneuron-like cell-derived EVs was evaluated by expression analysis of the pro-inflammatory interleukin 1β (IL-1β) and interleukin-6 (IL-6) (Figure 5), and anti-inflammatory interleukin 4 and interleukin 10 (IL-4, IL-10) genes, measured as Ct (cycle threshold) and ∆∆Ct values (Figure 6). After the statistical analysis, our results showed that the pro-inflammatory cytokines were significantly more expressed in the BV2 cells exposed to small EVs (sEVs) derived from mSOD1 (mutant superoxide dismutase) NSC-34 motoneuron-like cells vs. SOD1WT-NSC-34 motoneuron-like cells. After 12 h of exposition to sEVs derived from mSOD1-NSC-34 MN-like cells, we measured a high level of IL-1β mRNA expression, 5.89 ± 0.28, 3.73 ± 0.2, 4.94 ± 0.3, 5.29 ± 0.26 fold change vs. wtSOD1-NSC-34 MN-like cells for mSOD1A4V, mSOD1G37R, mSOD1G85R, mSOD1G93A, respectively (Figure 5A). After exposing the cells to sEVs derived from mSOD1-NSC-34 motoneuron-like cells for 24 h, we observed a decrease in IL-1β mRNA levels (Figure 5B). However, following an additional 24 h of culture, there was a notable overexpression of IL-1β and IL-6 across all SOD1 mutations analyzed, as illustrated in Figure 5C. In particular, in BV2 cells challenged with sEVs derived from mSOD1A4V, mSOD1G37R, mSOD1G85R, and mSOD1G93A cells, we measured an increase in IL-1β transcript levels (4.01± 0.2, 16.14 ± 0.75, 17.62 ± 0.85, 20.65 ± 0.98 fold change vs. SOD1WT cells, respectively). Likewise, we measured an increase in IL-6 transcript levels equal to 4.86 ± 0.26, 37.67 ± 1.6, 15.49 ± 0.77, and 20.94 ± 1.0 fold change vs. SOD1WT cells in BV2 cells exposed to sEVs derived from mSOD1A4V, mSOD1G37R, mSOD1G85R, and mSOD1G93A cells, respectively (Figure 5C). BV2 cell exposure to large extracellular vesicles (lEVs) and vesicle-deprived conditioned medium (free EVs CM) does not affect the relative expression of IL-1β and IL-6 mRNA at any time point (Figure 5).

The anti-inflammatory cytokines interleukin 4 and interleukin 10 (IL-4 and IL-10) promote the polarization of microglia cells in a neuroprotective phenotype [38,39,40] and can modulate the phagocytic activity of microglia [41]. The exposure of BV2 cells to sEVs derived from mSOD1-NSC-34 motoneuron-like cells induces overexpression of anti-inflammatory cytokines. Specifically, the IL-4 gene exhibits an overexpression of 1.39 ± 0.08, 4.03 ± 0.2, 3.48 ± 0.16, and 7.01 ± 0.36 fold change compared to SOD1WT cells when BV2 cells were exposed for 24 h to mSOD1A4V, mSOD1G37R, mSOD1G85R, and mSOD1G93A-derived sEVs, respectively (Figure 6B). No regulation in IL-4 expression is observed after 12 or 48 h of exposure (Figure 6A,B). Regarding IL-10 gene expression, we observed an increase in the BV2 cells exposed to lEVs derived from NSC-34 motoneuron-like cells after 24 and 48 h (Figure 6B,C); no regulation is observed after 12 of exposure (Figure 6A). BV2 cell exposure to free EVs CM does not affect the IL-4 and IL-10 mRNA relative expression at any time point considered (Figure 6).

## 4. Discussion

A plethora of studies highlight the pivotal role of extracellular vesicle (EVs)-mediated signalling in various brain pathologies, including cancer [35] and inflammatory [42] and neurodegenerative diseases [22]. Among them, Amyotrophic Lateral Sclerosis (ALS) represents a persistent neurodegenerative disease predominantly targeting the spinal cord, bulbar, and corticospinal motor neurons (MNs), and the primary indicators encompass developing muscle atrophy, and paralysis, culminating in fatality within five years post-disease inception. Recent studies have affirmed the significance of intercellular communication between MNs and microglia in ALS pathogenesis, which can be disrupted even before the onset of ALS symptoms [43]. The latest research is mainly focused on the discovery of soluble biomarkers for neurodegenerative disease, and, in this field, EVs are promising candidates as they are mediators of cell-to-cell communication [44] in the central nervous system (CNS), where EVs facilitate the transmission of signalling molecules between neurons, thereby influencing their development and functionality [45].

In this study, we analyzed the involvement of EVs derived from mSOD1-NSC-34 MN-like cells in the regulation of microglial polarization. In this regard, the BV2 cells were incubated for 12, 24, and 48 h in the presence of the EVs derived from mutant superoxide dismutase 1 (mSOD1)-NSC-34 cells; at the end of each exposure time, we analyzed 1) the state of microglial inflammation through RT-PCR of IL-1β, IL-6, IL-4, and IL-10, 2) the expression of proteins involved in inflammasome activation (IL -β and caspase 1), cell death (caspase 3), glial cell recruitment (CXCR1), and, finally, 3) the presence of the TGFβ cytokine receptor (TGFβ-R2).

To this purpose, we choose the NSC-34 cell line, an immortalized and proliferative hybrid cell line obtained from the spinal cord and neuroblastoma, as a cellular in vitro model to investigate the physiopathological mechanisms of ALS, since this cell line is indicated in a review of 2023 by Zhou [46] as a good cellular model of ALS, even if a manuscript by Hounoum in 2016 indicated that the motor neuron-like NSC-34 cell line is not a suitable in vitro model to study glutamate-induced excitotoxicity and to explore the pathogenesis of glutamate-mediated excitotoxicity at the cellular level in ALS and other motor neuron diseases [47]. In [46], several compelling reasons to utilize NSC-34 cells as an in vitro model for neurological diseases are reported. Firstly, this cell line is derived from a homogeneous population of MNs, making it particularly well-suited for investigating neurodegenerative disorders that target MNs, such as ALS. Additionally, NSC-34 cells are capable of surviving and preserving their phenotype for extended time in vitro, rendering them ideal for studying progressive diseases. Furthermore, these cells are easily subject to genetic manipulation, such as introducing specific mutations or knocking down particular genes, enabling researchers to explore the effects of these alterations on disease progression [46].

As reported in our previous manuscript, these cells (i) show a neuronal phenotype and metabolism after differentiation with DMEM/Ham’s F12 containing retinoic acid 1 μM; (ii) present a high transfection efficiency (80%) with different mSOD plasmids (mSOD1wt, mSOD1A4V, mSOD1G37R, mSOD1G85R, mSOD1G93A); (iii) release a high amount of EVs which contain bioactive molecules [32].

The preliminary analysis has been the morphological characterization after 24 h to set up the experimental conditions. The microscope analysis suggested that BV2 cells cultured for 24 h with EVs or conditioned medium (CM) or vesicle-deprived conditioned medium (free EVs CM) of mSOD1 NSC-34 cells display different amounts of three phenotypes, i.e., quiescent, activated, or apoptotic. In particular, regardless of the type of mutation, BV2 cells cultured for 24 h with the CM show an activated and apoptotic cell phenotype, unlike BV2 cells cultured with the free EVs CM, which show proliferative and/or quiescent morphology. A high number of apoptotic figures is also visible in the treatment with sEVs, while the phenotype is a mix of quiescent and/or activated cells in the treatment with lEVs. Based on these results, we decided to set up our investigation from a molecular point of view taking into consideration several pathways of activation before and after 24 h, i.e., at 12 and 48 h of treatment, respectively, as the molecular activation occurs before the morphological signs and can disappear after morphological changes. We have never observed detached cells floating in the medium at 48 h of treatment.

Our first interest is to study the transforming growth factor-β2 (TGF-β2)-TGF-β type II receptor (TGFβ-R2)–CX3C chemokine receptor 1 (CX3CR1) signalling that regulates the maintenance of innate immunity in the brain by driving the activation of microglia [36].

The CX3CL1 (also known as fractalkine-FKN)–CX3CR1 signalling interaction between microglia and neurons plays a crucial role in both normal and diseased states. CX3CL1 exists as a membrane-bound or soluble molecule; the membrane-bound form is essential for cell adhesion and serves as an inhibitory signal for microglia. In various neurodegenerative conditions, including ALS, available findings are limited, making it challenging to define the precise function of FKN signalling. Moreover, there is a lack of consensus regarding the impact of fractalkine isoforms on pathological progression, and comprehensive insights into the significance of CX3CL1 and CX3CR1 in ALS are particularly scarce [43]. The disruption of communication between motor neurons and microglia due to the absence of the CX3CR1 receptor in SOD1G93A transgenic mice accelerates the progress of the disease and worsens neuronal loss, highlighting the protective function of CX3CR1 signalling which is able to modulate the survival and course of ALS [48]. In physiological states, neurons exhibit FKN expression on their cellular surface and consistently release this molecule into the surrounding microenvironment where it is recognized by microglial CX3CR1 receptors. Activation of CX3CR1 typically supports the maintenance of microglial cells in a quiescent state, but can also stimulate their movement, thereby enhancing neuronal function stability and serving as a neuroprotective mechanism. Nevertheless, FKN could also trigger microglial activation and contribute to neurotoxic outcomes [49]. As summarized in Table 1, our results show a low expression of CX3CR1 in BV2 cells at all culture times and the SOD1 mutation type considered excepted an increase after 24 h of culture in the presence of mSOD1-G93A-derived lEVs. This suggested to us that probably mSOD1 NSC-34 MN-like cells do not release FKN, and, consequently, microglia are activated. Moreover, we can speculate that mSOD1-G93A-derived lEVs could transport FKN on their surface.

This finding is corroborated by results achieved regarding TGFβ-R2 expression (Table 1). TGF-β is a key signalling molecule involved in regulating the inflammatory response in glial cells. Research has shown that TGF-β can both promote and inhibit glial inflammation, depending on the specific context and cellular environment. The investigation carried out by Travis unveiled that TGFβRI and TGFβ-R2 are prominently abundant in microglia, in contrast to splenic macrophages [50]. The TGF-βII/TGFβ-R2 signalling pathway substantially influences the suppressive impact of NG2 glial cells in the mature mouse brain by enhancing SMAD2 phosphorylation, thereby upregulating CX3CR1 expression [36]. In our hands, the high expression of TGFβ-R2 downregulates the activation of CX3CR1 expression in BV2 cells challenged for 24 and 48 h with sEVs and lEVs derived from mSOD1 NSC-34 cells, except for mSOD1-G93A, which induces an upregulation of both TGFβ-R2 and CX3CR1 expression.

The low expression of TGFβ-R2 after 12 h of both EV and free EVs CM exposure of BV2 cells results in a consequent increase in IL-1β. This effect is particularly evident when BV2 cells were challenged with free EVs CM of mSOD1-NSC-34 cells, suggesting that soluble molecules are also involved in this signalling. Moreover, at 48 h of exposure to EVs, the high levels of TGFβ-R2 down-regulate levels of IL-1β expression, permitting us to speculate that mSOD1 NSC-34 cells release TGF-β2 in culture medium both as soluble molecules and packaged EVs (Table 1).

Interleukin-1β (IL-1β), a member of the IL-1 cytokine family, is produced as an inert pro-IL1β that undergoes proteolytic cleavage by caspase-1 that, upon exposure to diverse danger signals, assembles with intracellular proteins to form the inflammasome [51]. The active form of caspase-1 is detected in the cerebrospinal fluid and spinal cord specimens of patients with ALS as well as in an ALS mouse model [52]. In 2018, Lang suggested that the macrophage Migration Inhibitory Factor (MIF) is also involved in the activation of the inflammasome, regulating, in this manner, the release of IL-1α, IL-1β, and IL-18 [37].

MIF is a potent pro-inflammatory cytokine widely expressed in the CNS where it is produced by most cell types in the brain, including microglia, astrocytes, and neurons. It promotes the production of other immune mediators contributing to persistent glial activation, and this results in consequent chronic neuroinflammation and neurodegeneration contributing to CNS disorders [53].

According to these sentences, we observed, in BV2 cells, an overexpression of caspase 1 after 12 and 24 h of exposure to EVs, and an overexpression of MIF in line with the expression increase of IL-1β. The culture of BV2 cells with free EVs CM does not show expression of caspase 1 and MIF, corroborating our morphological observation that suggests no presence of dead cells. Surprisingly, the analysis of caspase 3 activation, while confirming the early (12 h) activation of inflammasome upon culture of BV2 cells with mSOD1 NSC-34 cells by an overexpression of caspase 3 only at 48 h, does not confirm the morphological observation of BV2 cultured in the free EVs CM of mSOD1 NSC-34 cells. In fact, an early expression (12 h) of caspase 3 has been measured for all mSOD1 mutation types considered, except mSOD1G93A. The presence of activated caspase 3 disappeared at 48 h (Table 1). We speculated that free EVs CM containing only soluble molecules released by mSOD1 NSC-34 cells induces cell death through a pathway not involving inflammasome activation and that the morphological evidence of dead cells will appear later than 24 h. In fact, caspase 3 is an effector caspase able to activate death by catalyzing the specific cleavage of many key cellular proteins leading to morphological features of apoptosis, such as blebbing and/or condensation and fragmentation of chromatin [54,55,56].

Pasinelli describes the progression of caspase activation in ALS linked to mutant SOD1 observed in both cell cultures and transgenic mouse models of ALS. In mutant SOD1 mice, caspase-1 becomes active early in the disease process, several months before neuronal death, while, in cell cultures, activation follows promptly after exposure to oxidative stress. Activation of caspase-3 takes place after caspase-1 activation [57], which is also noted in our results.

The analysis of pro- (IL-1β and IL-6) and anti-inflammatory (IL-4 and IL-10) cytokine expression showed an early polarization of microglia toward an M1 pro-inflammatory phenotype that shifts to a late M2 anti-inflammatory one in an experimental condition-dependent manner. These data also indicate that the response of immune cells to EVs released by mSOD1 NSC-34 cells depends on immune cell type, as previous experiments performed by us demonstrated that, unlike in microglia, the response of macrophages to EVs produced by mSOD1 NSC-34 cells starts as an anti-inflammatory polarization and ends as a pro-inflammatory polarization [30]. Here, the acute inflammation mediated by the expression of IL-1β at 12 h of culture in the presence of mSOD1 NSC-34 cell-derived lEVs switches towards chronic inflammation through the expression of IL-6. Finally, we observed an increase in IL-10 mRNA levels of BV2 when cells were exposed for 24 and 48 h to mSOD1 NSC-34-derived lEVs. Conversely, we measured an increase in IL-4 transcripts in all experimental conditions only after 24 hours of culture, i.e., culture in the presence of sEVs, lEVs, and free EVs CM. This suggests that sEVs induce in BV2 cells an M1 polarization, while lEVs polarize BV2 cells toward an M2 phenotype, and free EVs CM induces only an anti-inflammatory response via IL-4 expression. Our data are in agreement with the findings of Pinto, suggesting the switch to mixed M1 and M2 subpopulations in the N9 microglial cells treated with exosomes derived from mutant SOD1 (G93A) NSC-34 cells [33]. Gravel suggests that the elevated IL-10 concentrations found in microglia during the initial stages of ALS serve as a regulatory and compensatory adaptive immune strategy, functioning as a non-neuronal factor influencing the clinical initiation of the disease [58] and a promising therapeutic strategy in halting microglia activation and associated effects in MN degeneration.

## 5. Conclusions

In conclusion, the data obtained demonstrate a polarization of microglial cells exposed to mSOD1 NSC-34 motoneuron-derived EVs towards an early pro-inflammatory neurotoxic phenotype which, therefore, can participate in motor neuron degeneration and a late anti-inflammatory neuroprotective status with the appearance of a mixed M1 and M2 microglia subpopulations. In particular, the response of BV2 cells depends on the type of SOD1 mutation present in NSC-34 cells and is strictly dependent on mSOD1 NSC-34 EV type. In particular, sEVs modulate the inflammatory status of microglia cells more than lEVs. Conversely, the expression of TGFβ RII, CX3CR1, MIF, caspase 1, and caspase 3 is mainly regulated by lEVs. This suggests a complex network of signalling that should be deeply investigated to understand the impact of immune cell polarization on MN damage, as the exact MN death trigger remains indeterminate and the primary events instigating pathology remain a subject of debate.

## Figures and Tables

**Table 1:** Effect of vesicle-deprived conditioned medium (free EVs CM), small EVs (sEVs), and large EVs (lEVs) of mSOD1 NSC-34 MN-like cells on the expression of proteins involved in inflammasome activation (IL-β and caspase 1), cell death (caspase 3), glial cell recruitment (CXCR1), and presence of the TGFβ cytokine receptor (TGFβ-R2), in BV2 cells cultured for 12, 24, and 48 h.

		12 h			24 h			48 h		
		sEVs	lEVs	Free EVs CM	sEVs	lEVs	Free EVs CM	sEVs	lEVs	Free EVs CM
TGFβRII	A4V	+2	-	-	+3	-	-	+2.1	+7.6	-
	G37R	+2.2	-	-	+3.2	-	-	+3.2	+6	-
	G85R	-	-	-	+2.8	-	-	+2.2	+17.6	-
	G93A	+1.9	+2	-	+2.5	+13.7	-	+2.5	+6.8	-
CX3CR1	A4V	-	-	-	-	-	-	-	-	-
	G37R	-	-	-	-	-	-	-	-	-
	G85R	-	-	-	-	-	-	-	-	-
	G93A	-	-	-	-	+3.5	-	-	-	-
IL-1β	A4V	+2.8	-	+13.6	-	-	-	+2.1	-	-
	G37R	-	-	+4.2	+ 3.4	+2.2	-	-	-	-
	G85R	-	+2	+21.9	-	+2.1	-	-	-	-
	G93A	-	+2.2	+5.9	+ 1.9	+11.1	-	-	-	-
MIF	A4V	-	-	-	-	-	-	-	-	-
	G37R	-	-	-	-	+3.1	-	-	-	-
	G85R	-	-	-	-	+10.8	-	-	-	-
	G93A	+2.2	+2.2	-	-	+3	-	-	-	-
Caspase 1	A4V	-	-	-	-	+4.3	-	-	-	-
	G37R	-	+9.8	-	-	+8.9	-	-	-	-
	G85R	-	+11.6	-	-	+2.2	-	-	-	-
	G93A	-	+3.1	-	-	+4.1	-	-	-	-
Caspase 3	A4V	-	-	+3.4	-	-	-	-	-	-
	G37R	-	-	+2.9	-	+3.1	+3.6	-	-	-
	G85R	-	-	-	-	-	-	+1.8	+3.4	-
	G93A	-	-	+4.3	+ 2.1	-	+2.6	-	+3.2	-

Fold numbers of increase (+) or not modified (=) compared to the control (wt SOD1). TGFβ-RII (TGF-β type II receptor); CX3CR1 (CX3C chemokine receptor 1); IL1-β (interleukin 1β); MIF (macrophage inhibitory factor). Plasmids used for transfection: pF146 pSOD1WTAcGFP1 (Plasmid #26407); pF147 pSOD1A4VAcGFP1 (Plasmid #26408); pF148 pSOD1G37RAcGFP1 (Plasmid #26409); pF149 pSOD1G85RAcGFP1 (Plasmid #26410); pF150 pSOD1G93AAcGFP1 (Plasmid #26411).

## Data Availability

The original contributions presented in this study are included in the article/Supplementary Material; further inquiries can be directed to the corresponding authors.

## Supplementary Materials

The following supporting information can be downloaded at: https://www.mdpi.com/article/10.3390/biomedicines12092069/s1. Figure S1: Characterization of EVs derived from mSOD1 NSC-34 MN-like cells. Figure S2: Representative images of Western blot analysis of BV2 microglia cell lysates.
