# Supplementary material for: BV2 Microglial Cell Activation/Polarization Is Influenced by Extracellular Vesicles Released from Mutated SOD1 NSC-34 Motoneuron-like Cells
Source: Biomedicines. 2024 Sep 11;12(9):2069. doi: 10.3390/biomedicines12092069 (PMC11428949; doi:10.3390/biomedicines12092069)
Supplement: Supplementary file 1 [file biomedicines-12-02069-s001.zip › biomedicines-3156012-supplementary.pdf]

Figure S1

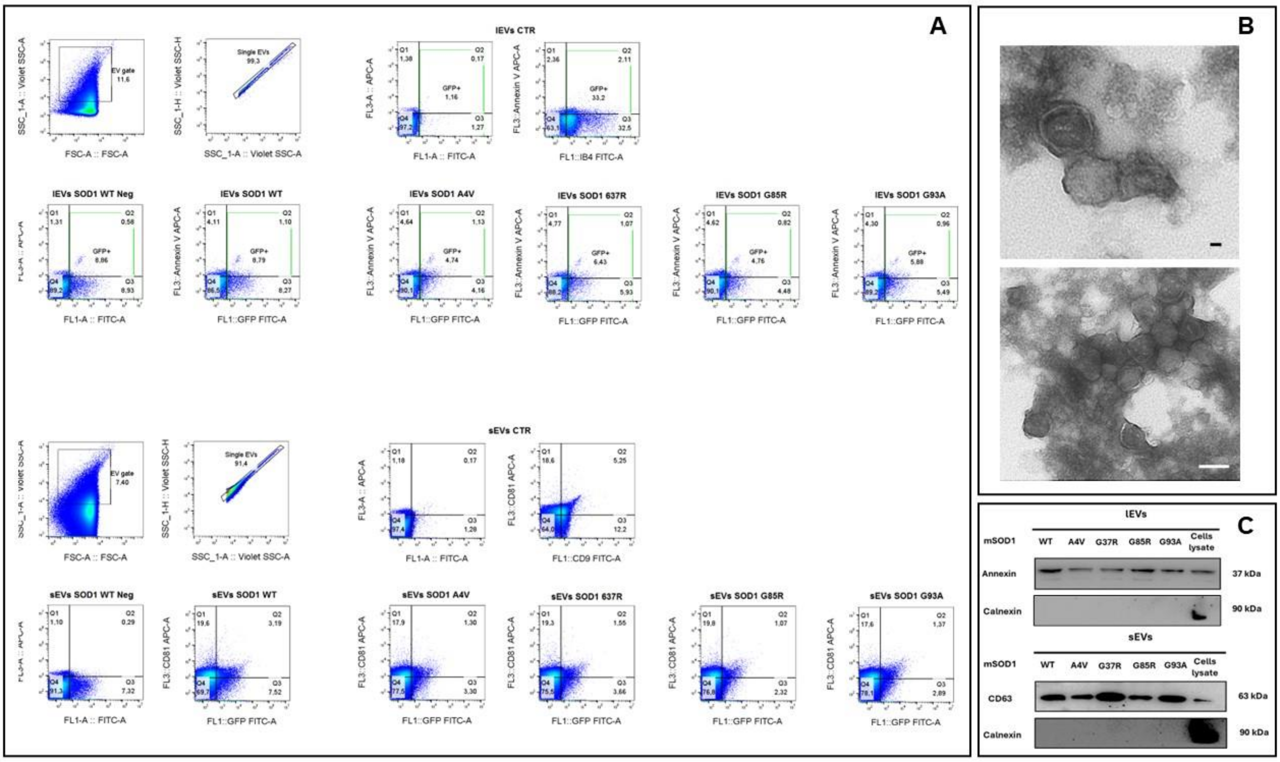

Characterization of EVs derived from mSOD1 NSC-34 MN-like cells. A: Quantitative analysis of large and small EVs isolated from mSOD1 NSC-34 MN-like cells using high-resolution flow cytometry. Flow cytometry gating strategy for phenotyping large and small EVs. Megamix-Plus FSC is used to set FSC-vSSC for EV events. APC-Annexin V and tetraspanin APC-CD81 are used to stain isolated GFP-positive lEVs and sEVs, respectively. Before analysis on a CytoFLEX S flow cytometer, EVs are diluted 1:200 in 1X PBS. For all assays, 30μL of control (unstained EV) and 30μL of each sample were measured at slow flow. B: Representative TEM images of lEVs (scale bar= 100 nm) and sEVs (scale bar= 100 nm) released from G93A mSOD1 NSC-34 MN-like cells. C: Representative membrane of large (Annexin A) and small (CD 63) EVs specific markers expression evaluated by Western Blotting analysis. Calnexin has been used to assess the absence of endoplasmic reticulum debris confirming the validity of the chosen isolation technique. Plasmids used for transfection: pF146 pSOD1WTAcGFP1 (Plasmid #26407); pF147 pSOD1A4VAcGFP1 (Plasmid #26408); pF148 pSOD1G37RAcGFP1 (Plasmid #26409); pF149 pSOD1G85RAcGFP1 (Plasmid #26410); pF150 pSOD1G93AAcGFP1 (Plasmid #26411). Antibodies: Anti- CD63 (# sc-365604, 1:50 dilution, Santa Cruz Biotechnology, Dallas, Texas, USA), anti-Annexin A1 (#sc-130305, 1:500 dilution; Santa Cruz Biotechnology, Dallas, Texas, USA), anti-Calnexin (#sc-23954, 1:500 dilution, Santa Cruz Biotechnology, Dallas, Texas, USA), anti β-actin (#MA1-91399, 1:2000 dilution; Invitrogen, Carlsbad, CA, USA).

Figure S2

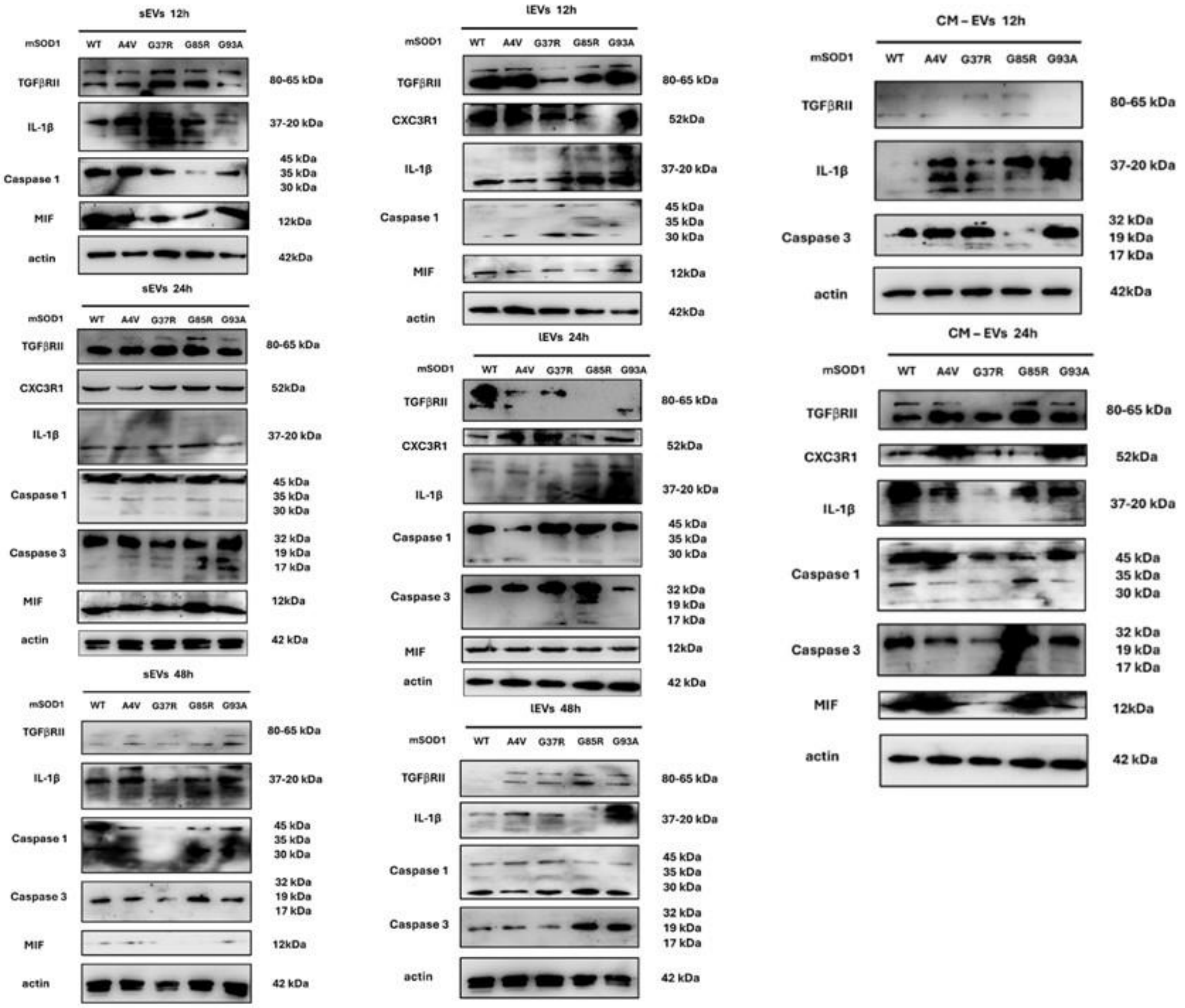

Representative images of Western blot analysis of BV2 microglia cell lysates. BV2 cells were cultured for 12, 24 and 48 hours in the presence of small EVs (sEVs), large EVs (lEVs) and vesicle-deprived conditioned medium (free EVs CM) of mSOD1 NSC-34 MN-like cells. The immunoreactive bands were detected by ChemiDoc Imaging System (Bio-Rad Laboratories, Hercules, CA, USA) by using a commercial enhanced chemiluminescence (ECL) reagent (Immobilon Crescendo Western HRP substrate; Merck Millipore, Darmstadt, Germany). β actin was used as control. TGFβ-RII (TGF-β type II receptor); CX3CR1 (CX3C chemokine receptor 1), IL1-β (Interleukin 1β), MIF (macrophage inhibitory factor). Plasmids used for transfection: pF146 pSOD1WTAcGFP1 (Plasmid #26407); pF147 pSOD1A4VAcGFP1 (Plasmid #26408); pF148 pSOD1G37RAcGFP1 (Plasmid #26409); pF149 pSOD1G85RAcGFP1 (Plasmid #26410); pF150 pSOD1G93AAcGFP1 (Plasmid #26411).
